# The impact of social media on self-esteem

**DOI:** 10.1192/j.eurpsy.2022.1410

**Published:** 2022-09-01

**Authors:** F.Z. Chamsi, I. Katir, A. Korchi, S. Belbachir, A. Ouanass

**Affiliations:** Psychiatric hospital Ar-razi, Psychiatry, Salé, Morocco

**Keywords:** self-esteem, social media, self-image, young population

## Abstract

**Introduction:**

The Social media have gained tremendous popularity over the past decade, these sites have occupied a major part of people’s lives, especially young people. Many teenagers use tik tok, instagram, snapchat and facebook, to build relationships, connect with the world, share and acquire knowledge and information, and build their personalities, their effects are not limited. to that, comparisons made using social networking sites have led people to have a drop in self-esteem, with all the complications that can cause (anxiety disorders, depression and the anxiety disorder , etc.)

**Objectives:**

Assessment of the impact of social media on the self-image, of young subjects in the Moroccan context

**Methods:**

Cross-sectional study with a descriptive and analytical aim, using a questionnaire and a satisfaction scale to assess the impact of social media on the self-image of young subjects in the Moroccan context. bibliographic research to objectify several studies on this subject

**Results:**

our results are close to the results of the literature. Sample of 200 young peoples was selected based on the confidence level of 80%. In order to test the hypothesis each respondent was given a questionnaire which tested their selfesteem and enquired the amount of time they spent on Facebook, instagram, tik tok, snapchat.

**Conclusions:**

social networks are a way to communicate information, ideas of ways of life. this communication includes harmful effects on the social behavior of young people

**Disclosure:**

No significant relationships.

